# A national survey of ambulance paramedics on the identification of patients with end of life care needs

**DOI:** 10.29045/14784726.2021.3.5.4.60

**Published:** 2021-03-01

**Authors:** Peter Eaton-Williams, Jack Barrett, Craig Mortimer, Julia Williams

**Affiliations:** South East Coast Ambulance Service NHS Foundation Trust; South East Coast Ambulance Service NHS Foundation Trust; South East Coast Ambulance Service NHS Foundation Trust; South East Coast Ambulance Service NHS Foundation Trust; Paramedic Clinical Research Unit (ParaCRU), University of Hertfordshire

## Abstract

**Aims:**

Developing the proactive identification of patients with end of life care (EoLC) needs within ambulance paramedic clinical practice may improve access to care for patients not benefitting from EoLC services at present. To inform development of this role, this study aims to assess whether ambulance paramedics currently identify EoLC patients, are aware of identification guidance and believe this role is appropriate for their practice.

**Methods:**

Between 4 November 2019 and 5 January 2020, registered paramedics from nine English NHS ambulance service trusts were invited to complete an online questionnaire. The questionnaire initially explored current practice and awareness, employing multiple-choice questions. The Gold Standards Framework Proactive Identification Guidance (GSF PIG) was then presented as an example of EoLC assessment guidance and further questions, permitting free-text responses, explored attitudes towards performing this role.

**Results:**

1643 questionnaires were analysed. Most participants (79.9%; n = 1313) perceived that they attended a patient who was formally unrecognised as within the last year of life on at least a monthly basis. Despite 72.0% (n = 1183) of paramedics indicating that they had previously made an EoLC referral to a General Practitioner (GP), only 30.5% (n = 501) were familiar with the GSF PIG and of those only 25.9% (n = 130) had received training in its use. Participants overwhelmingly believed that they can (94.4%; n = 1551) and should (97.0%; n = 1594) perform this role, yet current barriers were identified as the inaccessibility of a patient’s medical records, inadequate EoLC education and communication difficulties. Consequently, facilitators to performing this role were identified as the provision of further training in EoLC assessment guidance and establishing accessible, responsive EoLC referral pathways.

**Conclusion:**

Ambulance paramedics frequently encounter patients that they perceive are not receiving appropriate EoLC provision, and participants in this study overwhelmingly supported a role in highlighting this to primary care providers. Though many paramedics are already making referrals for these patients, the majority are performed without knowledge of validated EoLC assessment guidance. Provision of EoLC assessment training might therefore be expected to improve the timeliness and sensitivity of referrals, potentially addressing current inequalities in access to EoLC. The communication difficulties currently encountered when making a referral might be addressed by the provision of dedicated EoLC referral pathways. Future qualitative and quantitative evaluation of local initiatives providing both assessment training and referral pathways would be hugely beneficial for revealing the benefits and barriers associated with the development of this role in practice.

